# Trans-Activation between EphA and FGFR Regulates Self-Renewal and Differentiation of Mouse Embryonic Neural Stem/Progenitor Cells via Differential Activation of FRS2α

**DOI:** 10.1371/journal.pone.0128826

**Published:** 2015-05-29

**Authors:** Takahiro Sawada, Daiki Arai, Xuefeng Jing, Kenryo Furushima, Qingfa Chen, Kazuki Kawakami, Hideyuki Yokote, Masayasu Miyajima, Kazushige Sakaguchi

**Affiliations:** 1 Department of Molecular Cell Biology and Molecular Medicine, Institute of Advanced Medicine, Wakayama, Wakayama, Japan; 2 Laboratory Animal Center, Wakayama Medical University, Wakayama, Japan; Rutgers University, UNITED STATES

## Abstract

Ephs and FGFRs belong to a superfamily of receptor tyrosine kinases, playing important roles in stem cell biology. We previously reported that EphA4 and FGFR form a heterodimer following stimulation with ligands, trans-activating each other and signaling through a docking protein, FRS2α, that binds to both receptors. Here, we investigated whether the interaction between EphA4 and FGFRs can be generalized to other Ephs and FGFRs, and, in addition, examined the downstream signal mediating their function in embryonic neural stem/progenitor cells. We revealed that various Ephs and FGFRs interact with each other through similar molecular domains. When neural stem/progenitor cells were stimulated with FGF2 and ephrin-A1, the signal transduced from the EphA4/FGFR/FRS2α complex enhanced self-renewal, while stimulation with ephrin-A1 alone induced neuronal differentiation. The downstream signal required for neuronal differentiation appears to be MAP kinase mainly linked to the Ras family of G proteins. MAP kinase activation was delayed and sustained, distinct from the transient activation induced by FGF2. Interestingly, this effect on neuronal differentiation required the presence of FGFRs. Specific FGFR inhibitor almost completely abolished the function of ephrin-A1 stimulation. These findings suggest that the ternary complex of EphA, FGFR and FRS2α formed by ligand stimulation regulates self-renewal and differentiation of mouse embryonic neural stem/progenitor cells by ligand-specific fine tuning of the downstream signal via FRS2α.

## Introduction

Fibroblast growth factor receptor substrate 2α (FRS2α) is a major docking protein and mediator of signal transduction pathways downstream of the fibroblast growth factor receptor (FGFR) [1]. Stimulation with FGF ligands results in phosphorylation of multiple tyrosines on FRS2α, creating multiple binding motifs that recruit certain downstream signaling molecules. Four tyrosine phosphorylation sites on FRS2α bind to the adaptor molecule Grb2, while the remaining two bind to the Src Homology 2 (SH2) domain-containing tyrosine phosphatase, Shp2 [1]. Binding of Grb2 to FRS2α mediates not only the SOS-Ras-MAP kinase pathway, but also recruitment of an additional docking protein, Gab1, followed by activation of the phosphatidylinositol 3-kinase/Akt pathway, while binding of Shp2 mediates the Ras-MAP kinase signaling cascade [1, 2]. Binding of Shp2 to phosphorylated FRS2α also mediates formation and activation of the Shp2-Crk-C3G complex in response to NGF, leading to Rap1 activation followed by sustained MAP kinase activation [3–5]. Aoki et al. reported that Rap1, but not Ras, is activated by EphA receptors, mediating sustained MAP kinase activation in response to ephrin-A1 [6]. Shp2 binding sites of FRS2α appear to play an important role in maintaining neural stem/progenitor cells (NSPCs), while FGF2-induced activation of these sites mediates embryonic corticogenesis [7]. We previously reported that FRS2α forms a ternary complex with FGFR and EphA4 in response to stimulation with FGFs or ephrins, as well as mediating NSPC proliferation [8].

Eph receptors constitute the largest subfamily of the receptor tyrosine kinase superfamily [9]. Eph receptors and their ligands, ephrins, are likewise divided into two subclasses, type A and B, depending on their binding specificity. EphA receptors bind to the ephrin-A class of ligands, which are anchored to the cell membrane through glycosyl phosphatidyl inositol linkage, while EphB receptors bind to another class of ligands, ephrin-Bs, which have a transmembrane and short cytoplasmic domain. In general, EphAs bind to ephrin-As and EphBs to ephrin-Bs; however, cross-specificity has been reported in both the receptors and ligands. Interaction between Eph receptors and their membrane-bound ligands, ephrins, leads to contact-dependent bidirectional signaling into opposing cells, which regulates diverse developmental and physiological processes. Eph/ephrin signaling has multiple functions including cytoskeletal modulation affecting cell migration, growth cone repulsion and axon guidance [10], maintenance and plasticity regulation of neural stem cells in the subventricular niche [11], cell sorting during embryonic patterning [12], angiogenesis [13], bone homeostasis [14] and insulin secretion [15].

Our earlier discovery that EphA4 and FGFR form a heterodimer, trans-activating each other after stimulation with their ligands [16], led us to examine the function of this complex formation in NSPC proliferation and differentiation. Thus far, we have shown that FRS2α binds not only to FGFR but also EphA4 through distinct molecular regions as well as mediating differential downstream signals from the activated receptors. The signal depends on the ligand used for initial stimulation and, more or less, induces stem cell proliferation [8]. We also reported that the adult subventricular niche possesses a mechanism for regulation of both stem cell and angiogenic responses via ephrin-A1/EphA4-mediated signals [17]. Activation of EphA receptor-mediated signals by ephrin-A1 from within the lateral ventricle could potentially be utilized in the treatment of neurodegenerative diseases such as Parkinson’s disease. Another group revealed that EphA4 is expressed in adult neural stem cells in the subventricular zone, playing an important role in maintaining an undifferentiated state [18]. These findings suggested the need for further studies to determine what signals are responsible for self-renewal and differentiation of embryonic neural stem cells.

## Materials and Methods

### Reagents

Ephrin-A1 fused to human IgG(Fc) (ephrin-A1-Fc) was purchased from Sigma-Aldrich Co (St. Louis, MO, USA; Cat. #E9902). Before application, 5 μg of ephrin-A1-Fc was oligomerized via incubation with 12 μg of rabbit anti-human IgG(Fc) (Jackson ImmunoResearch Lab., West Grove, PA, USA; Cat. #309-005-008) in 1 ml of PBS at 4°C for at least 1 h. As a control, a human IgG(Fc) fragment (Jackson ImmunoResearch Lab.; Cat. #009-000-008) was used after oligomerization. FGFR inhibitor (SU5402; Cat. #572630), PI3-kinase inhibitor (LY294002; Cat. #440202) and MEK1/2 inhibitor (U0126; Cat. #662005) were purchased from Calbiochem (Billerica, MA, USA). The following antibodies were used according to the manufacturers’ instructions: mouse anti-myc antibody derived from hybridoma MYC1-9E10.2 (ATCC, Manassas, VA, USA; Cat. #CRL-1729), mouse anti-HA antibody derived from hybridoma 12CA5 (Roche, Mannheim, Germany; Cat. #11583816001), mouse anti-Flag M2 monoclonal antibody (clone M2; Sigma-Aldrich Co.; Cat. #F3165), mouse anti-HA monoclonal antibody (Santa Cruz Biotechnology, Santa Cruz, CA, USA; Cat. #sc-7392), rat anti-HA monoclonal antibody (clone 3F10; Roche, Cat. #11867423001), mouse anti-GFP monoclonal antibody (Santa Cruz Biotechnology, Cat. #sc-9996); rabbit anti-FGFR1 polyclonal antibody (Santa Cruz, Cat. #sc-121), rabbit anti-FGFR3 polyclonal antibody (Santa Cruz Biotechnology, Cat. #sc-123), rabbit anti-FRS2α polyclonal antibody (Santa Cruz Biotechnology, Cat. #sc-8318), rabbit anti-EphA4 polyclonal antibody (Santa Cruz Biotechnology, Cat. #sc-921), rabbit anti-Akt 1/2 polyclonal antibody (Santa Cruz Biotechnology, Cat. #sc-8312), rabbit anti-phospho-Akt (Ser473) monoclonal antibody (Cell Signaling Technology, Denvers, MA, USA; Cat. #4060), rabbit anti-phospho-p44/42 polyclonal mitogen-activated protein kinase (MAPK) (ERK) (Thr202/Tyr204) antibody (Cell Signaling Technology, Cat. #9101), rabbit anti-p44/42 MAPK (ERK) polyclonal antibody (Cell Signaling Technology, Cat. #9102), mouse anti-phosphotyrosine monoclonal antibody (clone 4G10, Millipore, Billerica, MA, USA Cat. #05–321) and rat anti-nestin antibody (Rat-401, DSHB, Iowa City, IA, USA). Alexa Fluor 488 and Alexa Fluor 568 were purchased from Life Technologies (Rockville, MD, USA).

### Ethics statement

All animal experiments were carried out in strict accordance with the recommendations of the Act on Welfare and Management of Animals (Law No. 105, Japan), the Fundamental Guidelines for Proper Conduct of Animal Experiment and Related Activities in Academic Research Institutions (Japanese Ministry of Education, Culture, Sports, Science and Technology, Notice No. 71, 2006) and the Standards Relating to the Care and Management of Laboratory Animals and Relief of Pain (Japanese Ministry of Environment, Notice No. 88, 2006). The protocol was approved by the Committee on the Ethics of Animal Experiments of Wakayama Medical University (Permit Number: 467). All surgeries were performed under sodium pentobarbital anesthesia with efforts made to minimize suffering.

### Cell culture

HEK293T cells were maintained in Dulbecco’s Modified Eagle’s Medium (DMEM) supplemented with 10% fetal bovine serum. Cells were transfected with eukaryotic expression vectors as described below. Prior to ligand stimulation, cells were preincubated in serum-free medium containing 0.5% bovine serum albumin (BSA) for 5 h. Chemical inhibitors, such as SU5402, were then added 1 h before completing the 5-h preincubation.

Mouse embryonic NSPCs were derived from the E14.5 telencephalon. Mouse embryos were obtained from a mated pregnant mouse which was euthanized by decapitation under sodium pentobarbital anesthesia. The whole brains were removed by decapitation from embryos and transferred into ice-chilled PBS in a culture-grade plate under the culture hood. The tissue region mainly including striatal telencephalon were isolated using micro-forceps under the stereomicroscope, then transferred into 1 ml of culture medium. Tissue pieces were dissociated into a single-cell slurry in the medium by pipetting up and down, and cells derived from 4 fetal brains were seeded into a T75 tissue culture flask. Cells were trypsinized and re-seeded into new flasks at an interval of 5 days.

Mouse NSPCs were maintained as neurospheres up to 2 passages in 50% DMEM/ 50% Ham’s F12 supplemented with 5 mM 4-(2-hydroxyethyl)-1-piperazineethanesulfonic acid (HEPES) buffer at pH 7.4, 0.6% glucose, 25 μg/mL insulin, 100 μg/mL transferrin, 20 nM progesterone, 60 μM putrescine, 30 nM sodium selenite, 20 ng/mL FGF2 and 20 ng/mL EGF as described previously [8]. For self-renewing assays, third passage cells cultured as neurospheres were trypsinized, dissociated mechanically by pipetting and seeded onto 96-well plates at 500 cells per well. In order to mark only the replicating cells, cells were transduced with a retrovirus carrying the expression cassette of enhanced green fluorescence protein (EGFP) and other molecules connected by the internal ribosomal entry site [19] at a multiplicity of infection of 5. They were incubated in medium containing different reagents in place of FGF2 and EGF. More than 95% of the retrovirus-infected cells expressed EGFP. NSPC self-renewal activity was measured according to the neurosphere formation assay [20, 21]. The number of neurospheres larger than 100 μm in diameter and positive for green fluorescence was counted under a fluorescent microscope within 24 h after 8-day incubation with the reagents. For stimulation with ligands, third passage sphere-cultured cells were preincubated for 4 h in medium devoid of FGF2 or EGF. Chemical inhibitors of certain signals were added 1 h after starting preincubation. Cells were lysed for protein analysis after incubation with the ligands.

### RNA extraction and reverse transcription-PCR

After 3-days culture, the neurospheres were rinsed with PBS. Total cellular RNA was then extracted using TRI reagent (Sigma-Aldrich Co.; Cat. #T9424). Reverse transcription (RT) was performed with M-MLV reverse transcriptase and an oligo(dT)20 reverse transcription primer to a final volume of 20 μl (SuperScript III; Invitrogen, Carlsbad, CA, USA; Cat. #18080–051). Polymerase chain reaction (PCR) was performed with 35 two-step cycles of 30 s at 94°C and 45 s at 58°C using 3 μl of RT product and GoTaq polymerase (Promega, Madison, WI, USA; Cat. #M7123). The PCR products were fractionated on 2% agarose gels, and bands stained with ethidium bromide and detected under ultraviolet irradiation. Primer sequences (forward and reverse) and product sizes were as described previously [17].

### DNA constructs, transfection, immunoprecipitation and immunoblotting

The eukaryotic expression vectors for wild-type (WT) and mutant EphA4, FGFRs and FRS2α were constructed as previously reported [8, 16]. Transient transfection of HEK293T cells was performed via 48-h incubation with PerFectin (Genlantis, Cat# T303015, San Diego, CA) according to the manufacturer’s instructions. Cells were then used for stimulation with ligands following 5-h preincubation in serum-free medium. Immunoprecipitation and immunoblotting were performed according to the standard procedure [8, 16, 22]. We confirmed that anti-HA and anti-myc antibodies efficiently immunoprecipitate the tagged proteins in a protein dose-dependent manner. For immunoblotting analysis, antibodies were diluted in appropriate reagent at a dilution range of 1:1000 to 1:5000 according to the manufacturer’s instructions. All experiments were carried out at least twice to confirm reproducibility. Quantification of band intensity was carried out with digital data using the computer program CS Analyzer version 3.0 (ATTO Corporation, Tokyo, Japan).

### Ras and Rap1 activity assay

Ras and Rap1 activity in the mouse embryonic NSPCs in response to ephrin-A1 and FGF2 were measured using a Ras activation assay kit and Rap1 activation assay kit (Cell Biolabs, Inc., San Diego, CA, USA; Cat. #STA-400 and #STA-406-1), respectively. Active small G proteins isolated from each cell lysate were detected by immunoblotting and the band intensities evaluated using CS Analyzer version 3.0 (ATTO Corporation).

### Construction of retrovirus expression vectors

WT and mutant EphA4 and FRS2α were constructed in a pMXs-IG vector and incorporated into a retrovirus as previously reported [8, 16, 19, 22] via co-transfection of the pMXs-IG-based constructs with pCAGVSV-G, which encodes a vesicular stomatitis virus surface protein, under control of a chicken β-actin promoter.

### Immunocytochemistry

Cells cultured on chamber slides were fixed with 4% paraformaldehyde in PBS then permeabilized with 0.25% Triton X-100 in PBS mixed with 1% normal rabbit serum and incubated with primary antibodies at 4°C overnight. After washing in PBS, they were then incubated with secondary antibodies conjugated to Alexa Fluor 488 or Alexa Fluor 568 at 4°C for 2 h, followed by washing in PBS. Immunofluorescence was detected with a Keyence BZ-9000 microscope and the number of cells counted using the BZ-II analyzer included in the apparatus.

## Results

### Crosstalk between EphAs and FGFRs may have multiple functions in NSPCs

**Fig 1:**
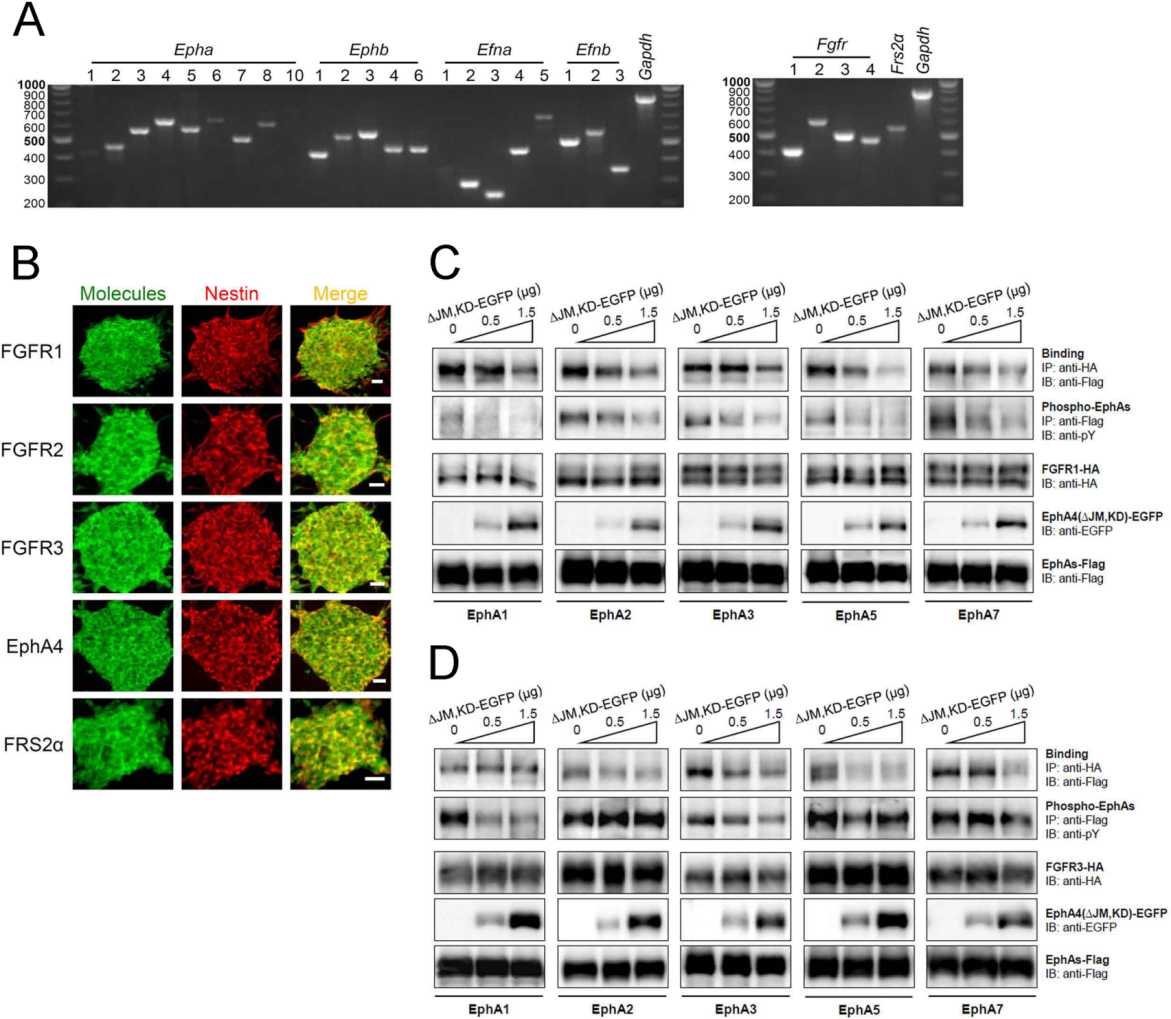
Expression and interaction of Ephs and FGFRs. (A) Expression of all Eph receptors, ephrin ligands, FGFRs and related molecules in mouse embryonic NSPCs. RT-PCR was performed with equal amounts of total RNA isolated from mouse NSPCs. Fragment lengths are indicated on the left in base pairs. (B) Co-expression of FGFRs, EphA4 and FRS2α (green, left panel), respectively, with the neural stem cell marker nestin (red, middle panel) in cultured neurospheres. Merged images are shown in right panels. Neurospheres were cultured on PLL-coated plates for a short time, fixed and immunostained. Immunofluorescent images were detected using a confocal microscopy with an appropriate optical filter. (C, D) Inhibition of FGFR-EphA binding with a dominant-negative EphA4 molecule, EphA4(ΔJM,KD), tagged with enhanced green fluorescence protein (ΔJM,KD-EGFP). FGFR1-HA (C) and FGFR3-HA (D) were co-expressed with Flag-tagged EphAs (EphA1, 2, 3, 5 and 7), respectively, and increasing doses of ΔJM,KD-EGFP in HEK293T cells. Binding of FGFR-HA with EphAs-Flag was examined with immunoprecipitation (IP) followed by SDS-PAGE and immunoblotting (IB) using the antibodies shown.

We investigated expression patterns of Eph receptors, ephrin ligands and other relevant signal molecules thought to regulate proliferation, self-renewal and differentiation of mouse embryonic NSPCs. Almost all Eph receptors and ephrin ligands were expressed in the NSPCs, and all FGFR family members were co-expressed (Fig 1A and 1B). First, we examined the interaction between EphAs and FGFRs via co-expression studies using HEK293T cells. All EphA members examined showed binding to both FGFR1 and FGFR3, while the EphA4 dominant-negative mutant EphA4(ΔJM,KD) inhibited binding in a dose-dependent fashion (Fig 1C and 1D). Trans-phosphorylation between EphA and FGFR was also inhibited by EphA4(ΔJM,KD). These results suggest that EphAs and FGFRs bind to each other through specific molecular regions, and moreover, that crosstalk is involved in various cellular and tissue functions when both kinds of receptor are expressed.

### Augmentation of NSPC self-renewal activity in response to FGF2 and ephrin-A1

**Fig 2:**
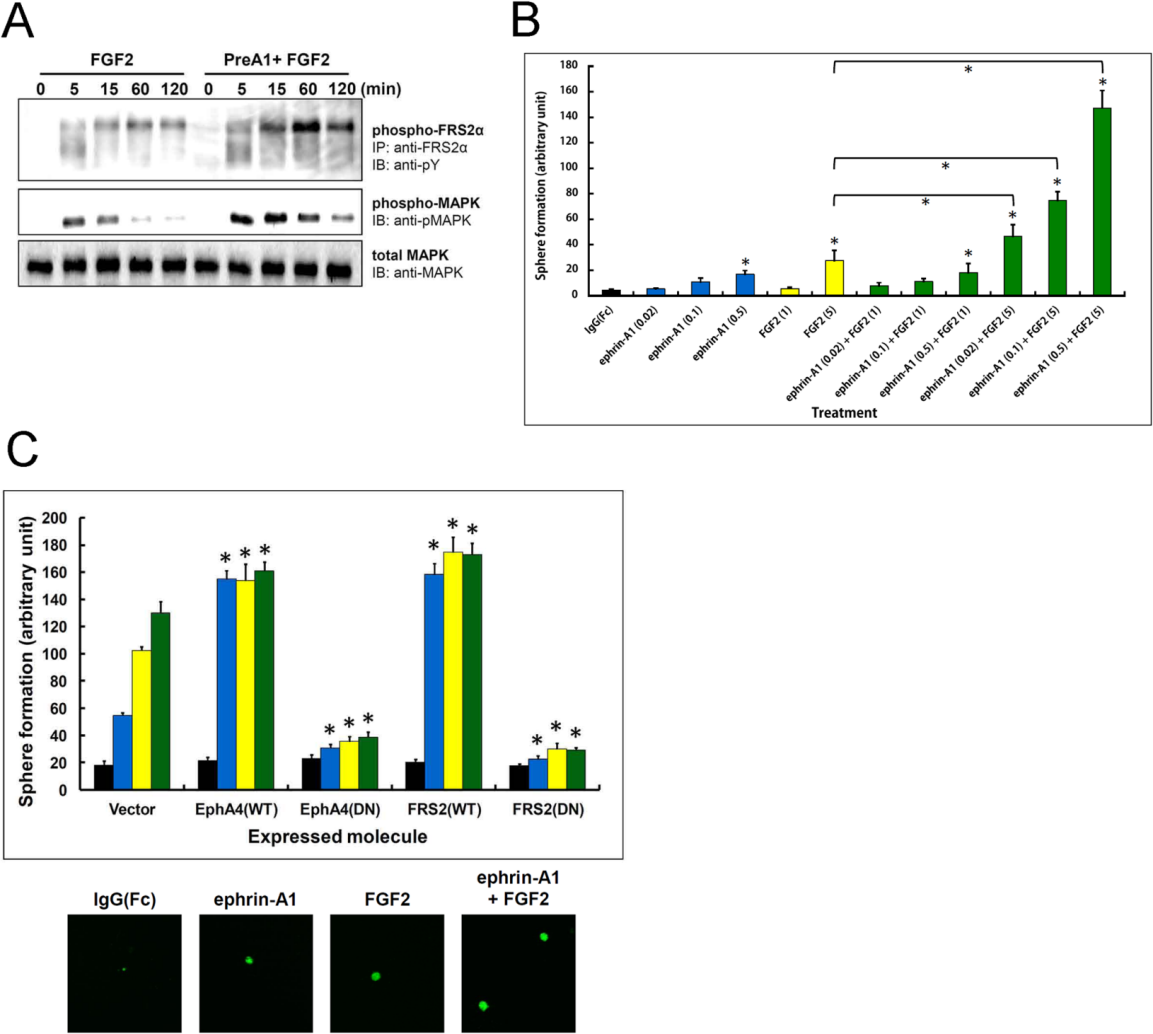
Effect of ephrin-A1 and FGF2 on NSPCs. (A) Tyrosine phosphorylation of FRS2α and MAP kinase in response to FGF2 stimulation with and without ephrin-A1 pre-treatment. Mouse NSPCs were treated with FGF2 (10 ng/ml) for the indicated periods of time with or without 45-min ephrin-A1 (0.5 μg/ml) pre-treatment (PreA1). FRS2α was immunoprecipitated using anti-FRS2α Ab from the cell lysate. Immunoprecipitates were fractionated by SDS-PAGE, and phosphorylated FRS2α was detected by immunoblotting with anti-pY Ab. For MAP kinase, cell lysates (50 μg of total protein per time point) were fractionated by SDS-PAGE and immunoblotted with anti-phospho-p44/42 MAPK (Thr202/Tyr204) Ab (phospho-MAPK) and anti-p44/42 MAPK Ab (total MAPK). (B) Synergistic effect of ephrin-A1 and FGF2 on neurosphere formation. Single NSPCs were plated 500 cells/well in 96-well plates for 3–5 days in medium containing different concentrations and combinations of reagent. Reagents were added repeatedly on the 3rd and 5th days and the number of spheres counted on the 10th day under a microscope. Concentrations of ephrin-A1 are expressed in μg/ml and that of FGF2 in ng/ml. Values were analyzed using Tukey’s multiple comparison test following ANOVA; n = 10 for each value. Bars represent the SD. * p<0.01 compared to the control treated with IgG(Fc) alone or between two treatment groups. (C) Self-renewing activity of NSPCs expressing EphA4(WT), EphA4(DN), FRS2(WT) and FRS2(DN), respectively. Study protocols were as in (B), but cells were exposed to vehicle (black), ephrin-A1 (0.5 μg/ml; blue), FGF2 (20 μg/ml; yellow), or a combination of ephrin-A1 and FGF2 (green). Bottom panels show micrographs of the spheres at the same magnification after various treatments of vector-expressing cells. Values were analyzed using Tukey’s multiple comparison test following ANOVA; n = 4 for each value. Bars represent the SD. * p<0.01 compared to controls transduced with the vector alone and treated with the same reagent. Values between WT- and DN-expressing cells were also significantly different (p<0.01) for each treatment except those treated with vehicle alone.

We previously reported that EphA4 forms a ternary complex with FGFRs and the docking protein FRS2α through distinct molecular regions. Stimulation with oligomerized ephrin-A1-Fc and/or FGF2 had differential effects on NSPC proliferation, with the combination of both having the maximum effect on mitogenic activity [8]. When NSPCs were stimulated with FGF2 (10 ng/ml) after 45 min pre-incubation with oligomerized ephrin-A1-Fc (0.5 μg/ml), they responded with sustained and enhanced tyrosine phosphorylation of FRS2α compared to FGF2 stimulation alone (Fig 2A). MAP kinase activation was also enhanced by addition of ephrin-A1, which strongly corresponded to the FRS2α phosphorylation results. To examine whether proliferating cells retain their stemness (multipotency), we examined the formation of secondary neurospheres as an indicator of self-renewal in response to varying concentrations of ephrin-A1 and/or FGF2. Respective treatment with ephrin-A1 and FGF2 resulted in dose-dependent augmentation of sphere formation activity; the increase with ephrin-A1 being smaller. Combined treatment with the starting doses of ephrin-A1 (0.02 μg/ml) and FGF2 (1 ng/ml) did not result in any significant increase compared with the vehicle (IgG(Fc)) alone. Meanwhile, treatment with 5 ng/ml FGF2 and varying concentrations of ephrin-A1 resulted in synergistic augmentation of sphere formation (Fig 2B). These results are consistent with our previous findings whereby a combination of ephrin-A1 and FGF2 synergistically augmented the proliferation of NSPCs.

We also examined functions of ternary complex formation in self-renewal activity. As shown in Fig 2C, both ephrin-A1 and FGF2 enhanced sphere formation, while a combination of the two increased it further in vector-transduced cells. Although retroviral transduction of WT *EphA4* and *FRS2α* respectively resulted in higher sphere formation activity in response to both ligands, transduction of the dominant-negative *EphA4* and *FRS2α* significantly inhibited sphere formation almost to the basal level, suggesting that EphA4 and/or FGFR signals play an important role not only in proliferation of NSPCs [8], but also in self-renewal. These results are consistent with our previous report whereby proliferating cells activated by EphA and FGFR signaling were shown to retain stemness.

### Ephrin-A1 induces neuronal differentiation rather than self-renewal

**Fig 3:**
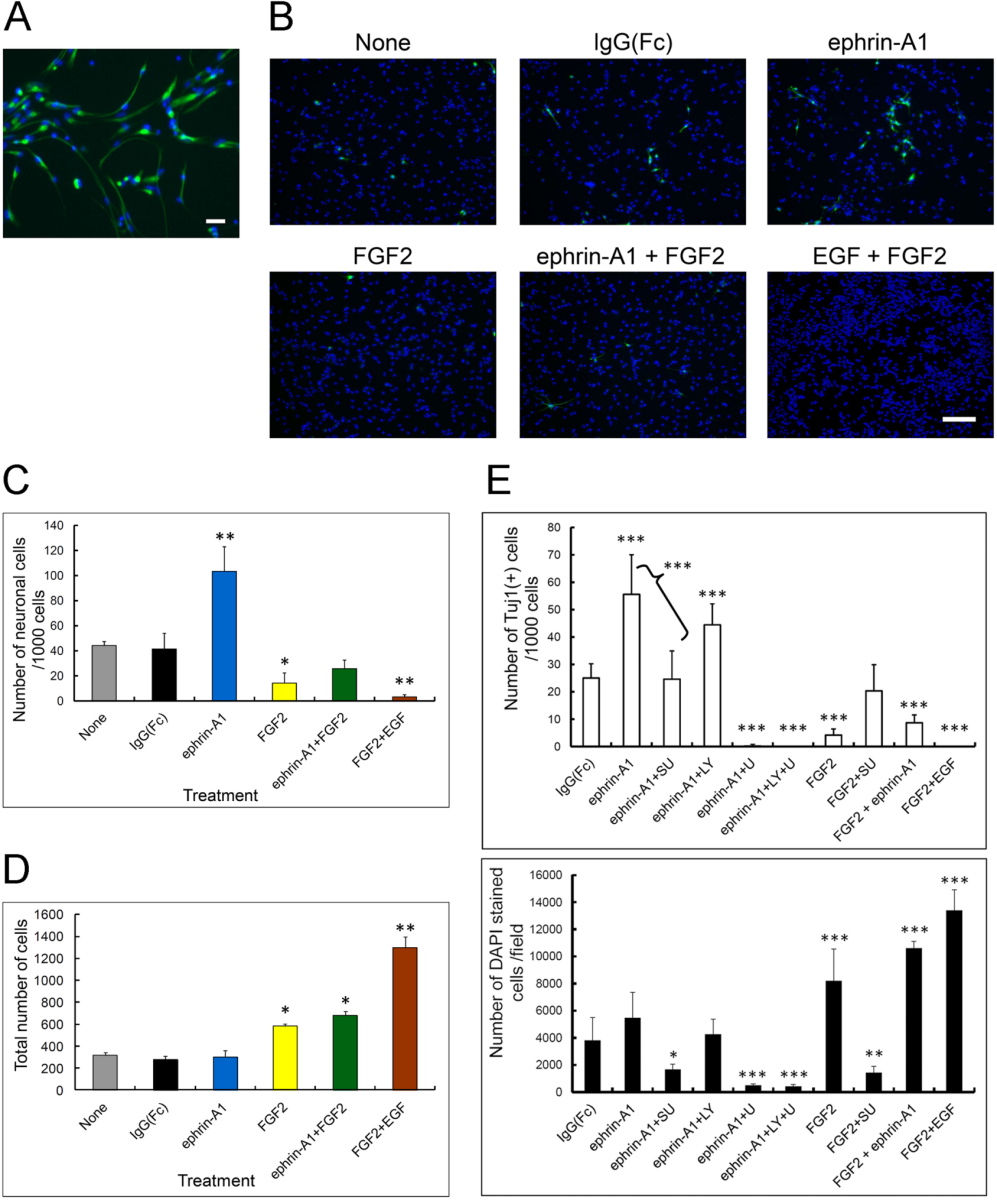
Differentiation and proliferation of mouse embryonic NSPCs induced by ephrin-A1 and/or FGF2 (A) NSPCs cultured as adherent cells. NSPCs were plated as adherent single cells at 1000 cells/well on a PLL- and fibronectin-coated cover glass in culture medium containing FGF2 (20 ng/ml) and EGF (20 ng/ml) for 3–5 days. Cells were then stained for anti-nestin (green), and counterstained with DAPI (blue) for nuclei. Scale bar: 50 μm. (B) NSPCs were plated as single cells 500 cells/well on PLL- and fibronectin-coated 96-well plates for 3–5 days in medium containing the different reagents. Reagents were re-added on days 3 and 5, and the numbers of Tuj1-positive cells counted between days 8 and 10 under an inverted fluorescent microscope with an optical filter. Scale bar: 50 μm. (C) The number of neuronally differentiated Tuj1-positive cells per 1000 cells. Values were analyzed using Tukey’s multiple comparison test following ANOVA (two-way). Bars represent the SD. * p<0.05, ** p<0.001 (n = 6) compared to the control. (D) The number of total cells in the area of one microscopic field. Values were analyzed using Tukey’s multiple comparison test following one-way ANOVA. Bars represent the SD. * p<0.05, ** p<0.001 (n = 6) compared to the control. (E) Effect of various signal inhibitors on the number of neuronally differentiated Tuj1-positive cells per 1000 cells (upper panel) and on the number of total cells in a single microscopic field (lower panel). NSPCs were plated as single adherent cells at 2 × 10^5^ cells/well on PLL- and fibronectin-coated 4-well chamber slides for 2 days in growth medium. Medium was replaced with growth factor-free medium containing ephrin-A1 and various inhibitors on day 2, and the number of Tuj1-positive cells and total cells per unit area counted on day 4 under an inverted fluorescent microscope with an optical filter. SU (SU5402), FGFR inhibitor; LY (LY294002), PI-3 kinase inhibitor; U (U0126), MEK1 and MEK2 inhibitor. Bars represent the SD. Values were analyzed using Tukey’s multiple comparison test following one-way ANOVA. * p<0.05, ** p<0.005, *** p<0.0001 (n>8) compared to the control (IgG(Fc)).

As shown in Fig 3A, immunofluorescent staining of nestin, a neural stem/progenitor cell specific marker, showed that all adherent cells cultured on a plate coated with poly L-lysine (PLL) and fibronectin retained characteristics of NSPCs in culture medium containing EGF and FGF2, even though they were spindle-like in shape. Using these adherent cells, we examined the effects of ephrin-A1 and/or FGF2 on differentiation of NSPCs.

To examine multipotency, we investigated the characteristics of cells stimulated with ephrin-A1 and/or FGF2. Immunofluorescent staining of the neuron specific marker Tuj1 in adherent cells cultured with ligands for 4 days revealed an increase in differentiation into neurons with ephrin-A1 treatment compared to treatment with control vehicles (none and IgG(Fc), respectively) (Fig 3B). Compared with combined treatment with FGF2 and ephrin-A1, single ephrin-A1 treatment resulted in a significant increase in the number of Tuj1-positive cells per total number of cells (Fig 3C). A small number of differentiated cells observed in negative controls (none and IgG(Fc), respectively) appeared to be the result of spontaneous neuronal differentiation caused by removal of growth factors (FGF2 and EGF) from the culture medium. Most Tuj1-negative cells in negative controls were positive for glial fibrillary acidic protein (GFAP)-positive (data not shown). The proliferative function of ephrin-A1 was almost zero compared with FGF2, FGF2 plus epnrin-A1 and FGF2 plus EGF treatment, respectively (Fig 3D). The slight self-renewal activity of ephrin-A1 alone shown in Fig 2C was possibly caused by differences in the culture method (sphere vs. adherent culture). We speculate that ephrin-A1 might be less effective at self-renewal under the adherent single cell culture system. These results demonstrate that ephrin-A1 is more involved in induction of neuronal differentiation rather than proliferation in NSPCs.

To verify the effect of ephrin-A1 itself on neuronal differentiation of NSPCs and to elucidate the signal pathways downstream of EphAs, we treated NSPCs with ephrin-A1 and inhibitors of various signaling pathways thought to be downstream of the ternary complex. Inhibition of the MAP kinase pathway with U0126 completely suppressed both the neuronal differentiation and proliferative activities induced by ephrin-A1 (Fig 3E). Treatment with PI3-kinase inhibitor LY294002 resulted in slight suppression of proliferation and differentiation compared with ephrin-A1 and vehicle (DMSO) treatment. Concomitant treatment with LY294002 and U0126 inhibited both proliferation and differentiation to a level similar to that with U0126 alone, suggesting that the effect was almost totally attributable to U0126. SU5402 is a specific inhibitor of FGFR; however, ephrin-A1-induced proliferation and differentiation were suppressed by SU5402 almost to the level of FGF2 plus SU5402 treatment. These results suggest that MAP kinase activity is principally required for neuronal proliferation and differentiation, and that trans-activation of FGFRs by EphAs plays an important role in these processes.

### Differential phosphorylation of FRS2α by EphA4 and FGFR1

**Fig 4:**
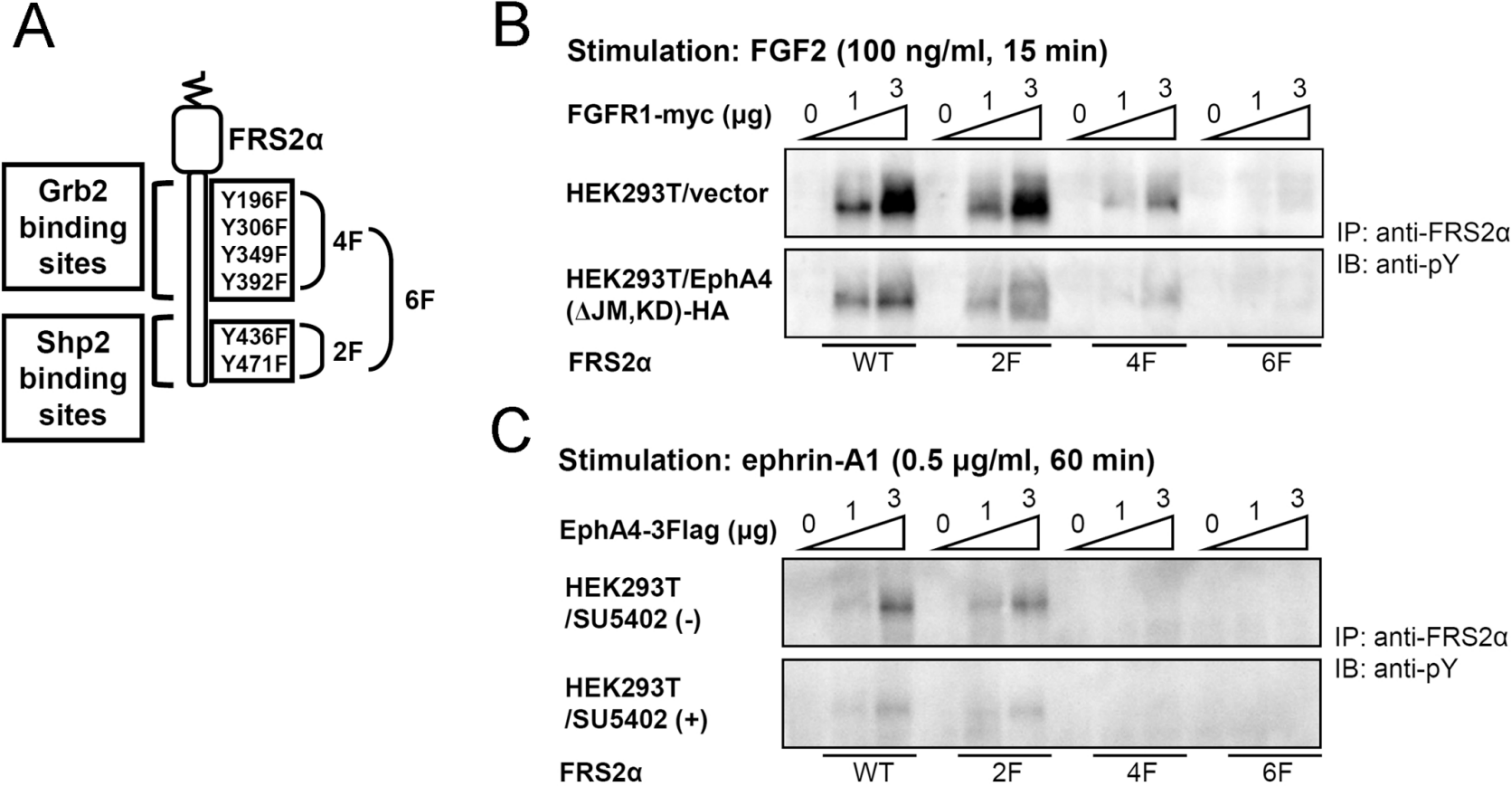
Differential phosphorylation of tyrosines in FRS2α. (A) Schematic representation of FRS2α mutants ectopically expressed in HEK293T cells. Mutant 4F contains phenylalanine in place of four tyrosine residues (Y169, Y306, Y349 and Y392), and is devoid of Grb2 binding. Similarly, mutant 2F contains phenylalanine in place of two tyrosine residues (Y439 and Y471), and is devoid of Shp2 binding. Mutant 6F carries the mutations of both 4F and 2F. (B, C) Tyrosine phosphorylation of FRS2α via FGFR1 and EphA4, respectively. Increasing doses (0, 1 and 3 μg per 60-mm plate) of pcDNA3.1/FGFR1-Myc or pcDNA3.1/EphA4-3Flag were co-transfected with pcDNA3.1/FRS2α (WT or mutants, 4 μg/plate) into HEK293T cells. A dominant-negative EphA4, EphA4(ΔJM,KD)-HA, was ectopically expressed in some HEK293T cells using a retrovirus vector (B, bottom panel). Some transfected cells were pretreated with 25 μM of SU5402 for 1 h before exposure to ephrin-A1 (C, bottom). The transfected cells were stimulated with FGF2 (100 ng/ml) for 15 min (B) or ephrin-A1 (0.5 μg/ml) for 60 min (C). In separate experiments, the maximal effects of FGF2 and ephrin-A1 were shown to occur at 15 and 60 min, respectively.

The signal mechanisms of the ternary complex comprising EphAs, FGFRs and FRS2α are yet to be clarified with regards to function. We previously reported that activated EphA4 directly binds to and phosphorylates FRS2α [8]. In the present study, we examined phosphorylation target sites of FRS2α with EphA4 kinase. To do so, we used WT and various mutants of FRS2α. The mutants, in which tyrosine residues were replaced by phenylalanine residues, included FRS2α-2F (a mutant of Shp2 binding sites), FRS2α-4F (a mutant of Grb2 binding sites) and FRS2α-6F (a mutant of both Shp2 and Grb2 binding sites) (Fig 4A). Co-expression studies of these FRS2α mutants in HEK293T cells with increasing doses of FGFR1 revealed that both Shp2 and Grb2 binding sites are phosphorylated in a dose-dependent manner, suggesting that both are FGFR1 targets. Furthermore, phosphorylation of these tyrosines was reduced with exogenous overexpression of a dominant negative EphA4, EphA4(ΔJM,KD), suggesting that FGFR-dependent FRS2α phosphorylation is also partly dependent on EphA4 expression (Fig 4B). In contrast, co-expression of FRS2α with increasing doses of EphA4 resulted in tyrosine phosphorylation of Grb2-binding sites only in a dose-dependent manner (Fig 4C). Treatment with SU5402, a specific inhibitor of FGFR kinases, significantly reduced phosphorylation, although it did not abolish it completely. Thus, we concluded that both Shp2 and Grb2 binding tyrosines are FGFR targets, whereas Grb2 binding tyrosines are the major targets of EphA4.

### Signaling mechanisms of neuronal differentiation mediated by the EphA/FGFR/FRS2α ternary complex

**Fig 5:**
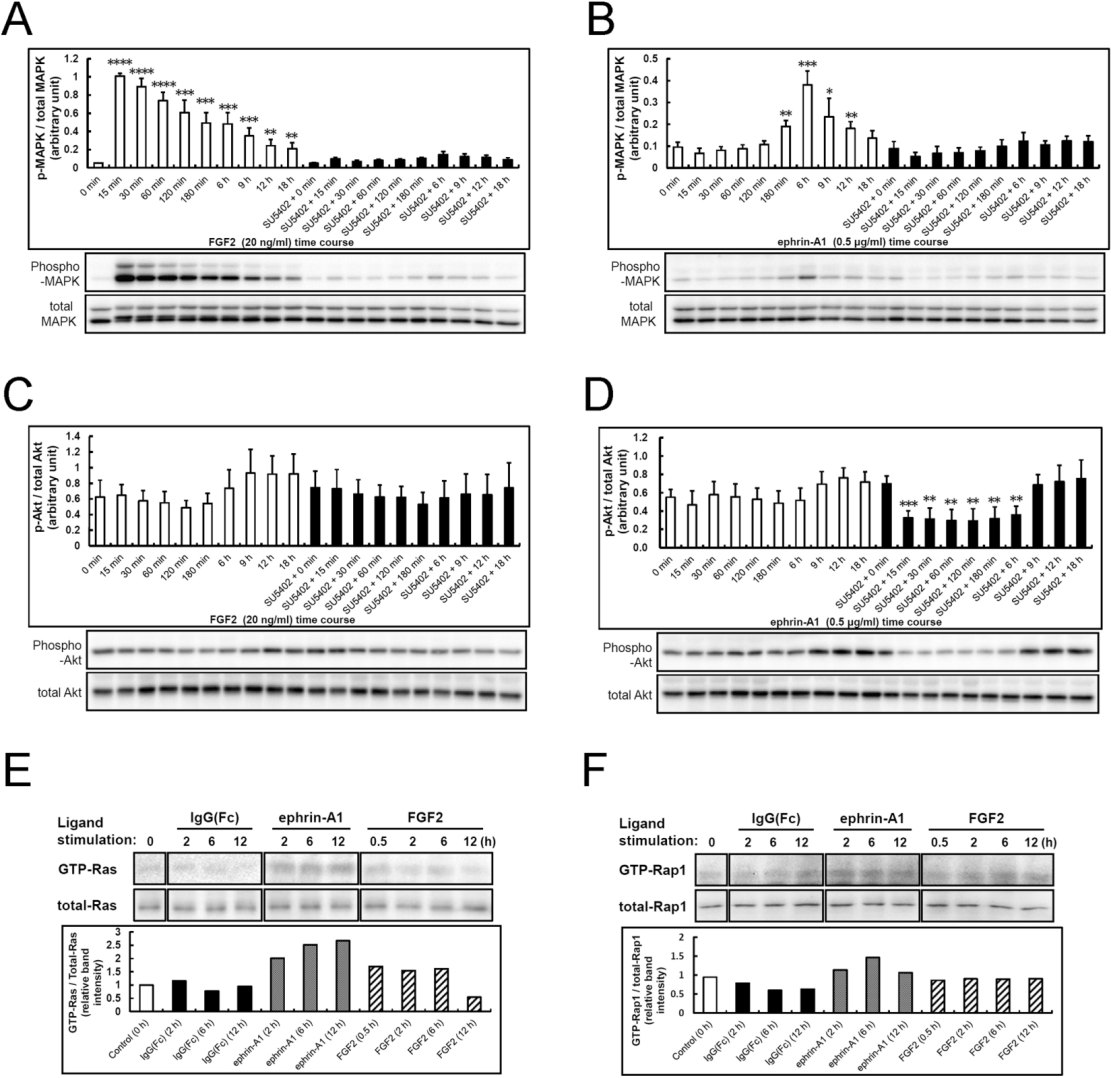
Ligand-specific activation of FRS2α, MAP kinase and Akt in mouse embryonic NSPCs. (A-D) Time course tyrosine phosphorylation of MAPK (ERK) and Akt in response to ephrin-A1 (0.5 μg/ml) and FGF2 (20 ng/ml), respectively, in mouse embryonic NSPCs. Cells were starved in growth factor-free medium containing 0.5% BSA and 20 mM HEPES buffer for 4 h. Activated phospho-MAPK (p-MAPK) and total amounts of MAPK (total MAPK) were detected by immunoblotting with anti-phospho-p44/42 MAPK (Thr202/Tyr204) and anti-p44/42 MAPK antibodies, respectively, using 20 μg of cell lysate for each time point. Phospho-Akt (p-Akt) and the total amount of Akt (total Akt) were also detected by immunoblotting with anti-phospho-Akt and anti-Akt antibodies, respectively. SU5402 (final concentration of 25 μM) was added 3 h before exposure to the ligand. Activation of MAP kinase and Akt were quantified in four different experiments (bottom: representative blotting result) and values were expressed as the ratio of p-MAPK/total MAPK or p-Akt/ total Akt. Bars represent the SD. * p<0.005, ** p<0.001, *** p<0.0001 (n = 4) compared to the value at the 0 time point in each treatment using the two-tailed Student’s t test. (E, F) Time course of Ras and Rap1 activation in response to ephrin-A1 (0.5 μg/ml) and FGF2 (20 ng/ml), respectively, in mouse embryonic NSPCs. Experiments were carried out twice with similar results. One of the blots is shown. Mean values of active (GTP-bound) and total Ras and Rap1 under various conditions were evaluated with the ratio of GTP-bound Ras/total Ras and GTP-bound Rap1/total Rap1, respectively.

The functions of Grb2 in mediating activation of the PI3-kinase-Akt pathway and SOS-Ras-MAP kinase pathway are well known. Since MAP kinase and PI3-kinase signals have been implicated in cell proliferation and differentiation activities, respectively, we examined their activities in NSPCs. Time-course analysis of FGF2 stimulation showed quick and robust activation of MAP kinase, which was suppressed almost completely by SU5402 (Fig 5A). In contrast, ephrin-A1 stimulation resulted in delayed weaker activation of MAP kinase, peaking approximately at 6 h, which was also suppressed by SU5402 (Fig 5B). In contrast, Akt activity did not change significantly from the basal level (0 min) following application of both FGF2 and ephrin-A1, even though a slight increase was observed at later time points between 9 and 18 h (Fig 5C and 5D). Pre-treatment with SU5402 did not affect Akt activity following FGF2 stimulation, but reduced Akt activity following ephrin-A1 stimulation at early time points (15 min-6 h). It was previously reported that activation of EphA2 via ephrin-A1 causes attenuation of Akt phosphorylation [23]. Since NSPCs also express EphA2 (Fig 1A), it is possible that the suppression of Akt activity with SU5402 pre-treatment might have had an effect on ephrin-A1/EphA2 signaling. That is, SU5402 might attenuate Akt phosphorylation. These results suggest that the delayed weak ephrin-A1-induced MAP kinase activation in NSPCs is caused by trans-phosphorylation of FGFR via active EphA receptor kinases, probably mediated mainly by the FRS2α-Grb2/SOS-Ras pathway. These findings also suggest why ephrin-A1-induced neuronal differentiation was suppressed by SU5402 (Fig 3D).

Subsequently, we performed further analysis of the delayed weak MAP kinase activation in response to ephrin-A1. To examine the activation status of downstream molecules of FRS2α in response to ephrin-A1 and FGF2 in NSPCs, activities of small G proteins, Ras and Rap1, were measured by pull-down assay. Ephrin-A1 increased the amount of GTP-bound Ras and sustained it even 12 h later, whereas the effect of FGF2 attenuated after 6 h of stimulation (Fig 5E). Ephrin-A1 slightly increased the amount of GTP-bound Rap1, with a peak at 6h, although not as much as Ras activation (Fig 5B). FGF2 did not influence GTP-Rap1 (Fig 5F). These results suggest that ephrin-A1 causes delayed weak MAP kinase activation probably through activation of Ras, leading to neuronal differentiation.

In summary, we extensively studied signals of the EphA4/FGFR/FRS2α complex following stimulation with ephrin-A1. Co-activation of FGF and ephrin signals enhanced NSPC self-renewal. The findings further revealed that ephrin-induced NSPC neuronal differentiation is enhanced through trans-phosphorylation of FGFRs by EphA and subsequent delayed MAP kinase activation.

## Discussion

We previously reported that trans-activation between EphA4 and FGFRs is mediated by direct interaction of their cytoplasmic domains, which augments downstream signaling through activation of FRS2α [16]. We also reported that FRS2α directly binds to not only FGFRs but also EphA4, suggesting that FRS2α mediates the signal from the two receptors. The ternary complex composed of these three molecules mediates augmentation of NSPC proliferation in response to combined treatment with FGF2 and ephrin-A1 [8]. In a separate report, we also revealed that FGFR directly activates ephexin1, a downstream signal molecule of EphA4, which regulates migration and morphological changes in neuronal cells through modulation of Rho family GTPases [22].

**Fig 6:**
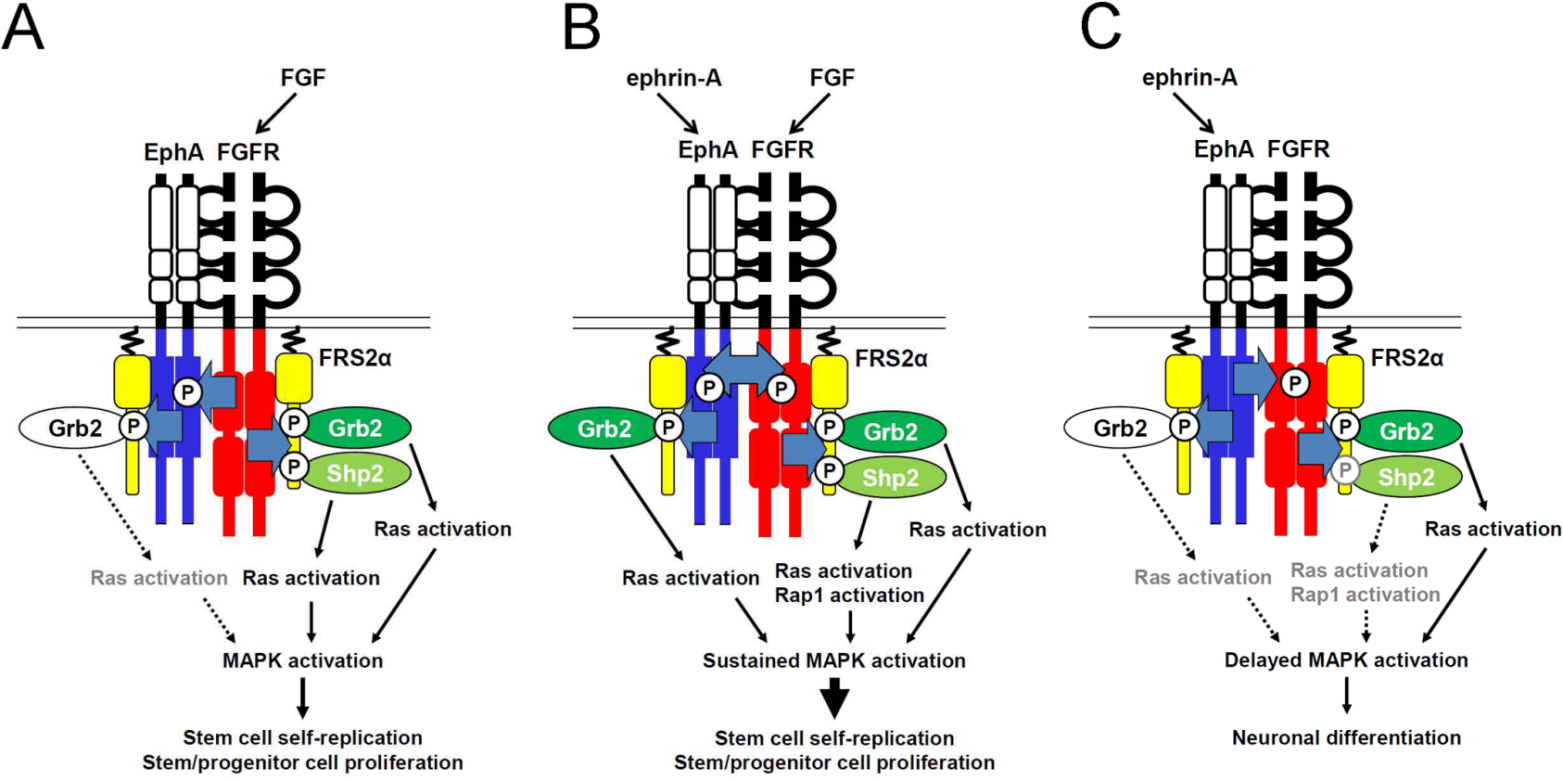
Schematic representation of EphA/FGFR/FRS2α complex signaling in NSPCs. (A) FGF2-induced activation of FGFR leads to phosphorylation of both Grb2 and Shp2 binding sites on FRS2α. Both the Shp2- and Grb2-mediated pathways were found to lead to activation of MAP kinase via activation of the Ras pathway. Rap1 was not significantly activated. (B) Co-stimulation with FGF2 and ephrin-A1 induced strong and sustained activation of FRS2α. Both the Ras- and Rap1-mediated pathways resulted in quick and robust MAP kinase activation, augmenting self-renewal of NSPCs. (C) Ephrin-A1-induced activation of EphA led to delayed weak activation of MAP kinase, and appeared to be mediated mainly by the Shp2-Ras and Shp2-Rap1 pathways via trans-phosphorylation of FGFR by EphA. Activation of Ras through the EphA-mediated Grb2-Ras pathway was weak. Dotted lines and grey letters indicate decreased signals compared with solid lines and black letters. Two FRS2α molecules are included in the complex to conveniently separate the signal transduction pathways mediated by ephrin/EphA4 and FGF/FGFR. The binding stoichiometry has yet to be studied.

In this report, we demonstrated that FGFRs bind to and phosphorylate almost all EphA members, and furthermore, that the interactions are inhibited by a dominant-negative form of EphA4. Consistent with our previous reports showing mitogenic activity, ternary complex signals were shown to mediate augmentation of NSPC self-renewing activity when stimulated with both FGF2 and ephrin-A1. The downstream signaling pathway of the ternary complex supporting augmentation is schematically shown in Fig 6. Ligand-stimulated FGFR resulted in phosphorylation of tyrosine residues on both the Grb2- and Shp2-binding sites of FRS2α, mediating activation of the Ras-MAP kinase pathway (Fig 6A), consistent with other reports [1, 2, 4]. In addition, combined stimulation with ephrin and FGF induced quick, robust and sustained MAP kinase activation caused by activation of both the Ras and Rap1 pathways. These signals resulted in augmentation of NSPC self-renewing activity (Fig 6B). The proliferating NSPCs retained the potential to differentiate into cells of neural lineage. Stimulation with ephrin-A1 alone activated MAP kinase signals, but in a delayed manner, and induced neuronal differentiation. This effect was suppressed by SU5402 (a specific inhibitor of FGFR kinase), suggesting that trans-activation of FGFRs plays an important role (Fig 6C). Rap1 was slightly activated consistent with the previous report [6]; however, did not appear to be the major signal downstream of the ephrin/Eph pathway.

We also determined the downstream signaling pathway using specific inhibitors. Treatment with LY294002 (PI-3 kinase inhibitor) did not result in a significant decrease in ephrin-A1-induced proliferation or differentiation, unlike U0126 (MEK1/2 inhibitor) treatment, which resulted in a significant decrease in both. These findings suggest that the MAP kinase pathway, but not the PI-3 kinase-Akt pathway, is involved in these effects. It was previously reported that FGF signaling through the MAP kinase pathway is required for neuronal differentiation and neural induction in zebrafish [24] and Xenopus [25], respectively. In PC12 cells, robust MAP kinase activation induced by EGF and FGF2, respectively, contributes to cellular proliferation, whereas nerve growth factor (NGF) stimulation leads to prolonged activation of MAP kinase through the FRS2α-Shp2-Rap1 pathway and to neuronal differentiation [3, 5, 26]. EphA appears to be a positive regulator of the Rap1-MAP kinase pathway that induces neurogenesis of mouse embryonic NSPCs [6]. These reports indicate that MAP kinase activity, which is regulated by EphAs or other growth factor receptors, is essential for neurogenesis. In this study, we demonstrated that Ras is the major G protein up-regulated by ephrin-A1 stimulation in mouse embryonic NSPCs. Taken together, these findings suggest that trans-activation of FGFRs via activation of EphAs mediates delayed MAP kinase activation principally through the FRS2α-Ras pathway, leading to neuronal differentiation of NSPCs.

Our study also shows that activation of the PI-3 kinase pathway does not play a major role in EphA-mediated neural stem cell differentiation. Inactivation of the PI-3 kinase/Akt pathway and activation of glycogen synthase kinase-3 β (GSK-3β) with differential application of FGF or EGF led to differentiation of human fetal neural stem cells into motor neurons *in vitro* [27]. Activation of the PI-3 kinase-Akt pathway is also necessary for PDGF-AA-induced differentiation of NSPCs into oligodendrocyte precursor cells (OPCs) [28]. However, in our study, ephrin-A1 stimulation resulted in significant attenuation of Akt activity. This might be attributable to crosstalk between EphA and Akt, as demonstrated by Miao et al. in glioma cells [23]. Ligand-stimulated EphA2 dephosphorylates a serine residue of Akt, which is conserved in EphA1 and EphA2. Attenuation of Akt phosphorylation might therefore be mediated by these same EphAs expressed in NSPCs.

In conclusion, we demonstrated that the ternary complex composed of EphA, FGFR and FRS2α mediates the self-renewing activity of mouse NSPCs in response to simultaneous stimulation with FGF2 and ephrin-A1. Stimulation with ephrin-A1 alone leads to delayed weak MAP kinase activation through characteristic phosphorylation of FRS2α via trans-activation of FGFR by EphA, resulting in induction of neuronal differentiation rather than proliferation and self-renewal. These findings suggest that the FGFR/EphA/FRS2α complex plays a critical role in switching between proliferation and differentiation of neural stem cells via domain-specific activation of FRS2α. Moreover, the findings provide important molecular evidence supporting ephrin-A1 stimulation of dopaminergic neurogenesis in a rat model of Parkinson’s disease [29].

## References

[1] Gotoh N. Regulation of growth factor signaling by FRS2 family docking/scaffold adaptor proteins. Cancer Sci. 2008;99: 1319–1325. 10.1111/j.1349-7006.2008.00840.x 18452557 PMC11159094

[2] Hadari YR, Gotoh N, Kouhara H, Lax I, Schlessinger J. Critical role for the docking-protein FRS2 alpha in FGF receptor-mediated signal transduction pathways. Proc Natl Acad Sci USA. 2001;98: 8578–8583. 11447289 10.1073/pnas.161259898 PMC37478

[3] Kao S, Jaiswal RK, Kolch W, Landreth GE. Identification of the mechanisms regulating the differential activation of the mapk cascade by epidermal growth factor and nerve growth factor in PC12 cells. J Biol Chem. 2001;276: 18169–18177. 11278445 10.1074/jbc.M008870200

[4] Wu C, Lai CF, Mobley WC. Nerve growth factor activates persistent Rap1 signaling in endosomes. J Neurosci. 2001;21: 5406–5416. 11466412 10.1523/JNEUROSCI.21-15-05406.2001 PMC6762651

[5] York RD, Yao H, Dillon T, Ellig CL, Eckert SP, McCleskey EW, et al Rap1 mediates sustained MAP kinase activation induced by nerve growth factor. Nature. 1998;392: 622–626. 9560161 10.1038/33451

[6] Aoki M, Yamashita T, Tohyama M. EphA receptors direct the differentiation of mammalian neural precursor cells through a mitogen-activated protein kinase-dependent pathway. J Biol Chem. 2004;279: 32643–32650. 15145949 10.1074/jbc.M313247200

[7] Yamamoto S, Yoshino I, Shimazaki T, Murohashi M, Hevner RF, Lax I, et al Essential role of Shp2-binding sites on FRS2alpha for corticogenesis and for FGF2-dependent proliferation of neural progenitor cells. Proc Natl Acad Sci USA. 2005;102: 15983–15988. 16239343 10.1073/pnas.0507961102 PMC1276098

[8] Sawada T, Jing X, Zhang Y, Shimada E, Yokote H, Miyajima M, et al Ternary complex formation of EphA4, FGFR and FRS2alpha plays an important role in the proliferation of embryonic neural stem/progenitor cells. Genes Cells. 2010;15: 297–311. 10.1111/j.1365-2443.2010.01391.x 20184660

[9] Kullander K, Klein R. Mechanisms and functions of Eph and ephrin signalling. Nat Rev Mol Cell Biol. 2002;3: 475–486. 12094214 10.1038/nrm856

[10] Shamah SM, Lin MZ, Goldberg JL, Estrach S, Sahin M, Hu L, et al EphA receptors regulate growth cone dynamics through the novel guanine nucleotide exchange factor ephexin. Cell. 2001;105: 233–244. 11336673 10.1016/s0092-8674(01)00314-2

[11] Nomura T, Goritz C, Catchpole T, Henkemeyer M, Frisen J. EphB signaling controls lineage plasticity of adult neural stem cell niche cells. Cell Stem Cell. 2010;7: 730–743. 10.1016/j.stem.2010.11.009 21112567 PMC3003830

[12] Xu Q, Mellitzer G, Robinson V, Wilkinson DG. In vivo cell sorting in complementary segmental domains mediated by Eph receptors and ephrins. Nature. 1999;399: 267–271. 10353250 10.1038/20452

[13] Wang HU, Chen ZF, Anderson DJ. Molecular distinction and angiogenic interaction between embryonic arteries and veins revealed by ephrin-B2 and its receptor Eph-B4. Cell. 1998;93: 741–753. 9630219 10.1016/s0092-8674(00)81436-1

[14] Zhao C, Irie N, Takada Y, Shimoda K, Miyamoto T, Nishiwaki T, et al Bidirectional ephrinB2-EphB4 signaling controls bone homeostasis. Cell Metab. 2006;4: 111–121. 16890539 10.1016/j.cmet.2006.05.012

[15] Konstantinova I, Nikolova G, Ohara-Imaizumi M, Meda P, Kucera T, Zarbalis K, et al EphA-Ephrin-A-mediated beta cell communication regulates insulin secretion from pancreatic islets. Cell. 2007;129: 359–370. 17448994 10.1016/j.cell.2007.02.044

[16] Yokote H, Fujita K, Jing X, Sawada T, Liang S, Yao L, et al Trans-activation of EphA4 and FGF receptors mediated by direct interactions between their cytoplasmic domains. Proc Natl Acad Sci USA. 2005;102: 18866–18871. 16365308 10.1073/pnas.0509741102 PMC1323220

[17] Jing X, Miwa H, Sawada T, Nakanishi I, Kondo T, Miyajima M, et al Ephrin-A1-mediated dopaminergic neurogenesis and angiogenesis in a rat model of Parkinson's disease. PLoS One. 2012;7: e32019 10.1371/journal.pone.0032019 22363788 PMC3282790

[18] Khodosevich K, Watanabe Y, Monyer H. EphA4 preserves postnatal and adult neural stem cells in an undifferentiated state in vivo. J Cell Sci. 2011;124: 1268–1279. 10.1242/jcs.076059 21444754

[19] Kitamura T, Koshino Y, Shibata F, Oki T, Nakajima H, Nosaka T, et al Retrovirus-mediated gene transfer and expression cloning: powerful tools in functional genomics. Exp Hematol. 2003;31: 1007–1014. 14585362

[20] Ahmed S. The culture of neural stem cells. J Cell Biochem. 2009;106: 1–6. 10.1002/jcb.21972 19021147

[21] Ferron SR, Andreu-Agullo C, Mira H, Sanchez P, Marques-Torrejon MA, Farinas I, et al A combined ex/in vivo assay to detect effects of exogenously added factors in neural stem cells. Nat Protoc. 2007;2: 849–859. 17474182 10.1038/nprot.2007.104

[22] Zhang Y, Sawada T, Jing X, Yokote H, Yan X, Sakaguchi K, et al Regulation of ephexin1, a guanine nucleotide exchange factor of Rho family GTPases, by fibroblast growth factor receptor-mediated tyrosine phosphorylation. J Biol Chem. 2007;282: 31103–31112. 17702745 10.1074/jbc.M704430200

[23] Miao H, Li DQ, Mukherjee A, Guo H, Petty A, Cutter J, et al EphA2 mediates ligand-dependent inhibition and ligand-independent promotion of cell migration and invasion via a reciprocal regulatory loop with Akt. Cancer Cell. 2009;16: 9–20. 10.1016/j.ccr.2009.04.009 19573808 PMC2860958

[24] Shinya M, Koshida S, Sawada A, Kuroiwa A, Takeda H. Fgf signalling through MAPK cascade is required for development of the subpallial telencephalon in zebrafish embryos. Development. 2001;128: 4153–4164. 11684653 10.1242/dev.128.21.4153

[25] Delaune E, Lemaire P, Kodjabachian L. Neural induction in Xenopus requires early FGF signalling in addition to BMP inhibition. Development. 2005;132: 299–310. 15590738 10.1242/dev.01582

[26] Sun P, Watanabe H, Takano K, Yokoyama T, Fujisawa J, Endo T, et al Sustained activation of M-Ras induced by nerve growth factor is essential for neuronal differentiation of PC12 cells. Genes Cells. 2006;11: 1097–1113. 16923128 10.1111/j.1365-2443.2006.01002.x

[27] Ojeda L, Gao J, Hooten KG, Wang E, Thonhoff JR, Dunn TJ, et al Critical role of PI3K/Akt/GSK3beta in motoneuron specification from human neural stem cells in response to FGF2 and EGF. PLoS One. 2011;6: e23414 10.1371/journal.pone.0023414 21887250 PMC3160859

[28] Hu JG, Wang YX, Wang HJ, Bao MS, Wang ZH, Ge X, et al PDGF-AA mediates B104CM-induced oligodendrocyte precursor cell differentiation of embryonic neural stem cells through Erk, PI3K, and p38 signaling. J Mol Neurosci. 2012;46: 644–653. 10.1007/s12031-011-9652-x 21953009

[29] Jing X, Miyajima M, Sawada T, Chen Q, Iida K, Furushima K, et al Crosstalk of humoral and cell-cell contact-mediated signals in postnatal body growth. Cell Rep. 2012;2: 652–665. 10.1016/j.celrep.2012.08.021 22999939

